# Physical activity and sports behavior of children and adolescents with and without disabilities from Germany: results from the cross-sectional KiGGS wave 2 study

**DOI:** 10.1186/s13690-025-01728-8

**Published:** 2025-09-16

**Authors:** Selina Seemüller, Anne Kerstin Reimers

**Affiliations:** Department of Sport Science and Sport, Friedrich-Alexander-Universität Erlangen-Nürnberg, Erlangen, Germany

**Keywords:** Inclusion, Youth, Impairment

## Abstract

**Background:**

Physical activity (PA) offers significant health benefits for children and adolescents with (CAWD) and without (CAWoD) disabilities. As there’s no data about the PA in different settings of CAWD in Germany, the present study aims to identify and compare PA participation in CAWD and CAWoD, focusing on meeting the World Health Organizational (WHO) PA recommendations and participation in sports activities (non-organised and organised sport in higher intensities) and sports clubs.

**Method:**

This study used data from the cross-sectional nationwide German Health Interview and Examination Survey for Children and Adolescents (KiGGS). To analyse and compare PA behaviour, Pearson Chi²-tests and binary logistic regression models were performed.

**Results:**

All PA values were analysed in 250 CAWD and 12,557 CAWoD aged 0–17 years. In CAWD PA values were lower than in CAWoD. Only 21.6% (54) of CAWD met the PA recommendations. Regarding participation in sport clubs, 44.6% (108) of CAWD compared to 60.3% (7,511) of CAWoD participated. Across sporting activities, 60.4% (151) of CAWD participate compared to 75.2% (9,488) of CAWoD. Statistically significant differences were identified across sport club participation (OR: 0.54, 95% CI [0.45, 0.78]) as well as sporting activities (OR: 0.59, 95% CI [0.42, 0.71]).

**Discussion:**

CAWD are less active compared to their peers without disabilities, showing significantly lower participation in sporting activities and sport clubs.

**Table:** 

**Text box 1. Contributions to the literature**
• CAWD are significantly less likely to participate in sports and sport clubs compared to children and adolescents without disabilities CAWoD, indicating a lack of inclusion and participation opportunities.
• There is no significant difference in meeting the World Health Organization (WHO) recommendations for physical activity between CAWD and CAWoD, though overall compliance is alarmingly low for both groups, highlighting the need for broader public health interventions.
• The study is one of the first using a large national sample to investigate sport (club) participation and compliance with WHO recommendations among German CAWD and CAWoD.

## Introduction

Physical activity (PA) is associated with numerous health benefits for people with and without disabilities [1, 2]. Low levels of PA can lead to lower cardiovascular fitness, impaired circulation as well as consequential diseases (e.g. obesity, osteoporosis) [3]. Further, physical inactivity can lead to psychosocial implications such as decreased social acceptance in children with (CAWD) and without disabilities (CAWoD) [4]. Especially in CAWD PA may offset predisposed risk for development of secondary health problems [5]. Being physically active increases health as well as participation and social interaction in CAWD [6]. Further, PA can increase motor development and can therefore increase independence [7].

To state a level of sufficient PA, the World Health Organization (WHO) published PA guidelines in 2010 for children and adolescents. As indicated by these guidelines children and adolescents should be active at least 60 min per day in moderate-to-vigorous PA (MVPA), especially aerobic activity [8]. According to the Global Matrix of Report Cards of 2018, only around one third of CAWoD achieved these WHO PA recommendations [9]. Regarding results of the Global Matrix of Para Report Cards, which is an international initiative that evaluates and compares PA levels and related behaviors of CAWD across different countries, only around 25% of CAWD achieve the recommendations [10]. A further national German KiGGS study indicated only 22.5% of CAWD fulfil the WHO recommendation [11]. The WHO updated their recommendations in 2020 and developed recommendations for CAWD. According to these recommendations CAWD should be active at least 60 min per day on average across the week [12]. The Global Matrix of Report Cards indicated, one third of CAWoD fulfil these new recommendations [13]. There’s no Global Matrix of Para Report Cards published using the WHO 2020 recommendations. Further, CAWD participate less in sports and exercise compared to CAWoD [14]. Less than 26% participate in organised sports and less than 39% participate in non-organised active play [10]. Compared to CAWoD these values are low. Regarding a recent review on PA in CAWD, PA levels varied widely [15] and makes it difficult to draw validated and representative conclusions. In Germany, a few studies have addressed PA in CAWD, such as the *AktiveKIDS* project, which collected data on PA and participation in children and adolescents with physical, intellectual, or visual impairments in a part of Germany [16, 17], and a study focusing on physical activity levels in children with visual impairments [18].

To the best of our knowledge, there is still no recent study in Germany that jointly examines both the achievement of the WHO PA recommendations and sport participation in organised and non-organised settings among CAWD with a wide variety of disabilities.

In Germany, disability is defined based on the German Social Code (Sozialgesetzbuch IX, SGB IX), which states that a person is considered disabled if there is a physical, mental, intellectual, or sensory chronic illness (longer than 6 months) that leads to participation restrictions of CAWD and CAWoD when the limited abilities due to the illness no longer allow to carry out the activities required by the ‘context’, (i.e. to fulfil role expectations) [19]. CAWD often face structural, environmental, and social barriers that limit their participation in PA, including accessibility issues, lack of inclusive sports programs, and lower social support [16, 20]. In Germany, children and adolescents typically access sports through school-based physical education and extracurricular programs as well as via local sports clubs, which form the backbone of the German sport system. While inclusive sports programs exist, they are not yet widespread and often depend on local initiatives or individual club structures. It is important to distinguish between participation in organized sports, such as those offered by sports clubs, and non-organized forms of physical activity, including informal active play or individual exercise. These forms differ in terms of access barriers, structure, social inclusion opportunities, and institutional support. Sports clubs often require membership, regular attendance, and may not offer inclusive environments for CAWD [21, 22], while informal PA settings may offer more flexibility but less social interaction or skill development [23]. Differentiating between these contexts enables a more accurate understanding of how CAWD engage in physical activity, and which settings may need targeted support or policy intervention. Despite these challenges, there is limited research on PA participation rates among CAWD in Germany, making it essential to examine their levels of activity in different settings.

Given the lack of comprehensive data on PA participation among CAWD in Germany, the present study aims to identify and compare PA participation in CAWD and CAWoD in Germany, focusing on meeting the WHO PA recommendations and participation in sports activities and sports clubs.

## Material and Methods

### Study design

The German Health Interview and Examination Survey for Children and Adolescents (KiGGS) is part of the Robert Koch Institute’s Federal Health Monitoring and is a German nationwide periodic cross-sectional survey on health status, health-related behaviours, and influencing factors of children and adolescents aged 0–17 years [24]. A stratified multi-stage probability sampling strategy with three levels was drawn to ensure a representative sample for the KiGGS [25]. First, a systematic sample of 167 German communities was randomly drawn using the Consulting - Information - Communication (BIK) classification system, which measures the degree of urbanisation and geographical distribution [24, 26]. In the next step, a sample of 28,400 randomly selected, age-stratified children and adolescents was drawn from the official population registers. KiGGS was conducted several times in the past starting in 2003. The most actual data is available from KiGGS wave 2 (2014–2017) in which a total of 15,023 children and adolescents (Response: 40.1%) participated [26]. From the total KiGGS Wave 2 sample, only participants with complete data on the relevant variables were included in the current analysis (final sample: *N* = 14,752). The Medical University of Hannover’s ethics committee approved the ethics of the KiGGS wave 2 (No. 2275 − 2014). The data is available at the Robert Koch Institute (RKI) in Germany. The authors requested the data set of certain variables for analysis purposes. Further information about the methodology of KiGGS can be read elsewhere [26–28].

### Data collection

Data collection in the cross-sectional KiGGS wave 2 took place between September 2014 and June 2017. In order to collect the data, three study teams carried out the examinations by constructing temporary examination centers in rented rooms at the 167 sample points. In order to reduce the influence of seasonal or regional factors, the order of the examinations at the sample points was systematically planned in a roadmap [29]. Besides medical examinations, for the cross-sectional KiGGS wave 2 a written, paper-based, and age-adjusted questionnaire on health and health-related behaviors was filled out by participants [27]. Children and adolescents aged at least 12 years answered the paper-based questionnaire by themselves, while in younger children, parents were asked to help them. All participants received written information and had to submit a written declaration of consent [27]. Participants who could not take part at the examination received a questionnaire by mail to replace the interview that would have been applied at the examination point [28]. To answer the present research questions only a few variables were requested from the RKI and sent to the authors for secondary data analysis purposes. These were the PA related variables as well as the disability-related variables and sociodemographic variables.

### Measures

#### Disability

Participants with disabilities were defined as all children and young people who have an officially recognised disability or are severely restricted in their ability to carry out everyday activities for more than six months due to illness. The following item was applied to differentiate between CAWD and CAWoD: “Do you have a disability officially recognised by the pension office?“. Answer options were “yes” or “no” [30].

#### Physical activity

We used three different outcomes for self-reported habitual PA: fulfilling the WHO PA recommendations (How many of the last 7 days have you been active for at least 60 min?), sports participations (Do you do sports? This refers to all types of sport in a club or outside a club, with the exception of sports lessons at school), sport club participation (Do you play sport at a sports club?). All PA outcomes were measured by dichotomous variables specifying whether the child or adolescent habitually participates in the PA setting or reaches the PA recommendation. For the item “fulfilment of WHO PA recommendations” the participants had to response they are active daily for at least 60 min. If the participants participated in sporting activities and participated in a sports club, they were given a “yes”. The calculation of the indicator for achieving the WHO PA recommendation (60 min of PA per day in moderate to vigorous intensity) was based on one item including sporting and everyday activities in a habitual week [31]. Further, “participation in sport clubs” was calculated on the self-reported physical activities in sport clubs with the minimum of one time per week habitually [32]. The same calculation was performed with the outcome “sport activities” [33].

### Confounding factors

Age and gender were treated as confounders in the present analysis. Further, residential area and residential location in Germany, as well as socio-economic status (SES) were included as confounders. Individual SES was recorded using an index based on information provided by parents on their level of education, occupational status and net household income [34]. All three SES domains were scored on a scale from 1 to 7 and combined into a composite score (range: 3–21), which was subsequently categorized into low (3.2– 8.7), medium (8.8–16.9) and high (17.0–21.0) socioeconomic status according to published guidelines [35]. Adolescents with separated parents were assigned the socioeconomic status of the parent they lived with.

### Statistical analysis

All analyses were performed with SPSS Version 29.01.0 [36]. Pearson Chi²-tests were used to test for significant associations between disability status and sociodemographic variables (gender, age group, and SES) to explore potential cumulative disadvantage. These analyses were performed solely for descriptive purposes prior to the regression analyses. Prevalence of PA levels in different PA variables (fulfilment of WHO PA recommendations, sporting activities, sport club participation) in CAWD and CAWoD were examined using descriptive statistics. In order to analyse differences in PA levels regarding CAWD and CAWoD, binary logistic regressions with the three dichotomous PA variables as dependent variables and age, gender, SES, residential area and initially also region in Germany as confounders were run. However, since region did not show any significant or meaningful associations with PA outcomes, it was excluded from the final models to improve interpretability. Age was classified into 0–3 years, 4–6 years, 7–12 years and 13–17 years. However, in the regression models, age was entered as a continuous variable (in years) to preserve statistical power and avoid information loss. The assumption of linearity for age was tested and met. Disability status was included as a binary independent variable (yes vs. no). Detailed categories of disability types are presented descriptively to characterize the sample but were not used in regression analyses. The residential area was created due to the number of inhabitants of the location, where the child/adolescent lived). Odds ratios (OR) were calculated from these binary logistic regressions. To reflect the main aim of the study, namely identifying potential disparities between CAWD and CAWoD, disability status was included as the key predictor in all regression models. The directionality of the associations (i.e., whether CAWD were more or less likely to meet PA thresholds) was interpreted post hoc and aligns with the descriptive results. For all analysis, the significance level was set to *p* < 0.05.

## Results

### Sample

For the current analysis data of 14,752 children and adolescents (7,349 boys, 7,304 girls) aged 0 to 17 years was eligible. On average, CAWD were 10.2 years old (SD = 4.1) and CAWoD were 9.2 years old (SD = 4.9). Ages were categorized into 0–3, 4–6, 7–12 and 13–17 years. Pearson-Chi² and Post-Hoc test revealed significant differences with regard to SES (X² [1] = 19.82, *p* < 0.001) and age (X² [1] = 51.16, *p* < 0.001). Specifically, the proportion of CAWD in the low SES category was higher (20.3%) compared to CAWoD (12.2%). A detailed description of the sample can be seen in Table 1.

**Table 1:** Description of the study sample

	Overall (*N* = 14,752)	Children with disabilities (*n* = 271)	Childen without disabilities (*n* = 14,481)	X²	df		*p*-value
Sex/gender [% (N)]				0.736	1		0.425
Boys	49.8 (7349)	52.4 (142)	94.8 (7207)				
Girls	50.2 (7403)	47.6 (129)	50.2 (7274)				
Age [% (N)]				51.156	17	< 0.001	
0–3 years	14.7 (2168)	4.4 (12)	14.9 (2156)				
Boys	7.4 (1087)	3.0 (8)	7.5 (1079)				
Girls	7.3 (1083)	2.2 (6)	7.4 (1077)				
4–6 years	18.0 (2659)	16.2 (44)	18.1 (2615)				
Boys	9.3 (1370)	7 (19)	9.3 (1351)				
Girls	8.7 (1289)	9.1 (25)	8.7 (1264)				
7–12 years	34.4 (5079)	41.7 (113)	38.5 (5556)				
Boys	16.0 (2363)	26.9 (73)	20.0 (2900)				
Girls	18.4 (2716)	22.1 (60)	18.4 (2656)				
13–17 years	30.0 (4421)	29.5 (80)	30.0 (4341)				
Boys	14.4 (2106)	15.5 (42)	14.3 (2064)				
Girls	15.8 (2315)	14.0 (38)	15.7 (2277)				
Socioeconomic status [% (N)]				19.821	2		< 0.001
Low	12.3 (1819)	20.3 (55)	12.2 (1764)				
Medium	61.2 (9031)	57.9 (157)	61.3 (8874)				
High	26.5 (3826)	21.8 (52)	26.5 (3774)				
Residential area [% (N)]				0.546	3	0.909	
Rural area	17.8 (2626)	16.2 (44)	17.8 (2582)				
Small town	32.3 (4759)	32.1 (87)	32.3 (4672)				
Medium-sized town	28.6 (4215)	29.6 (80)	28.6 (4138)				
city	21.3 (3152)	22.1 (60)	21.3 (3092)				
Region in Germany [% (N)]				2.124	2	0.346	
Former east	29.7 (4377)	31.7 (86)	29.6 (4291)				
Former west	66.8 (9855)	63.5 (172)	66.9 (9683)				
Berlin	3.5 (520)	4.8 (13)	3.5 (507)				
Type of disability [% (N)]							
Visceral	243	21.0 (57)					
Psychosocial		2.2 (6)					
Intellectual		32.1 (87)					
Sensorical		11.8 (32)					
Physical		22.6 (61)					
Degree of disability [% (N)]							
< 50%	232	21.1 (49)					
>=50%		78.9 (183)					

Note: df = degrees of freedom; N = total sample size; n = group sample size; *p* = *p*-value of the pearson chi-square statistic; rural area < 5,000 inhabitants; small town 5,000–20,000 inhabitants; medium-sized town 20,000-100,000 inhabitants; city > 100,000 inhabitants

### Physical activity participation in children with and without disabilities

Only 21.6% (54) of CAWD met the WHO PA recommendation of 60 min moderate to vigorous activity per day. Further, 60.4% (151) of CAWD participate in sporting activities and 44.6% (108) participate in sport clubs in their leisure time.

Compared to children and adolescents without disabilities (CAWoD), PA participation was consistently lower in CAWD across all domains. For example, 75.2% of CAWoD participated in sporting activities and 60.3% in sport clubs. Pearson-Chi² revealed statistically significant group differences in both sport participation (X² [1] = 28.68, *p* < 0.001) and sport club participation (X² [1] = 24.28, *p* < 0.001). Detailed results are presented in Table 2.

**Table 2:** Prevalence of physical activity participation in different domains

	Descriptive statistic			Bivariate analysis	
	Overall	Children with disability	Children without disability	T or X²	*p*-value
WHO-recommendations					
	*N* = 12,807	*n* = 250	*n* = 12,557		
Yes [% (N)]	25.5 (3264)	21.6 (54)	25.6 (3210)	2.03	0.154
No [% (N)]	74.5 (9543)	78.4 (196)	74.4 (9347)		
Sport participation					
	*N* = 12,863	*n* = 250	*n* = 12,613		
Yes [% (N)]	74.9 (9639)	60.4 (151)	75.2 (9488)	28.68	< 0.001
No [% (N)]	25.1 (3224)	39.6 (99)	24.8 (3125)		
Sports club participation					
	*N* = 12,699	*n* = 242	*n* = 12,457		
Yes [% (N)]	60.0 (7619)	44.6 (108)	60.3 (7511)	24.28	< 0.001
No [% (N)]	40.0 (5080)	55.5 (134)	39.7 (4946)		

Note: N = total sample size; n = group sample size

### Binary logistic regression analysis

The results of binary logistic regression are presented separately for each PA domain (Tables 3 and 4). The compliance with WHO PA recommendations, revealed no statistical difference in CAWD and CAWoD (OR: 0.86, 95% CI [0.63, 1.19])

**Table 3:** Binary logistic regression analysis of fulfilling the WHO recommendation

*N* = 14,752	B-weight	SE	X²	df	*P*	OR	95% CI (Lower – Upper)	
Disability	-0.146	0.163	0.809	1	0.368	0.86	0.6–1.2	
No Disability (ref.)						1.00		
Age	-0.174	0.005	998.953	1	< 0.001	0.84	0.8–0.9	
Sex								
Male	0.379	0.043	77.645	1	< 0.001	1.46	1.3 - 1.6	
Female (ref.)						1.00		
Socioeconomic status			11.677	2	0.003			
Low	0.175	0.074	5.594	1	0.018	1.19	1.0–1.4	
Middle	-0.053	0.050	1.101	1	0.294	0.95	0.9–1.0	
High (ref.)						1.00		
Residential area			31.106	3	< 0.001			
Rural area	0.157	0.072	4.803	1	0.028	1.17	1.0 - 1.3	
Small town	-0.145	0.064	5.115	1	0.024	0.87	0.8 - 1.0	
Medium-sized town	-0.150	0.066	5.258	1	0.022	0.86	0.8 - 1.0	
City (ref.)						1.00		

Note: B-weight = Beta Weight; SE = standard error; X² = Chi square; df = degrees of freedom; *p* = significance; OR = odds ratio; CI = confidence interval

**Table 4:** Binary logistic regression analysis of sport participation and sport club participation

Sports participation								
*N* = 14,752	B-weight	SE	X²	df	p	OR	95% CI (Lower - Upper)	
Disability	-0.608	0.138	19.384	1	< 0.001	0.55	0.4 - 0.7	
No Disability (ref.)						1.00		
Age	0.074	0.005	211.461	1	< 0.001	1.08	1.1 - 1.1	
Sex								
Male	0.158	0.042	14.044	1	< 0.001	1.17	1.1 - 1.3	
Female (ref.)						1.00		
Socioeconomic status			348.159	2	< 0.001			
Low	-1.334	0.072	347.665	1	< 0.001	0.26	0.2 - 0.3	
Middle	-0.570	0.055	107.838	1	< 0.001	0.57	0.5–0.6	
High (ref.)						1.00		
Residential area			15.285	3	0.002			
Rural area	0.267	0.073	13.285	1	< 0.001	1.31	1.1 - 1.5	
Small town	0.106	0.063	2.815	1	0.093	1.11	1.0 - 1.3	
Medium-sized town	0.057	0.064	0.784	1	0.376	1.06	0.9 - 1.2	
City (ref.)						1.00		
Sports Club participation								
*N* = 14,752	B-weight	SE	X²	df	p	OR	95% CI (Lower - Upper)	
Disability	-0.523	0.138	14.373	1	< 0.001	0.59	0.5 - 0.8	
Age	0.027	0.004	35.886	1	< 0.001	1.03	1.0 - 1.0	
Sex								
Male	0.233	0.038	38.007	1	< 0.001	1.26	1.2 - 1.4	
Female (ref.)						1.00		
Socioeconomic status			496.620	2	< 0.001			
Low	-1.509	0.68	495.048	1	< 0.001	0.22	0.2 - 0.3	
Middle	-0.558	0.046	145.048	1	< 0.001	0.57	0.5 - 0.6	
High (ref.)						1.00		
Residential area			19.460	3	< 0.001			
Rural area	0.275	0.065	17.893	1	< 0.001	1.32	1.2 - 1.5	
Small town	0.170	0.057	8.916	1	0.003	1.19	1.1 - 1.3	
Medium-sized town	0.106	0.058	3.400	1	0.065	1.11	1.0 - 1.2	
City (ref.)						1.00		

Note: B-weight = Beta Weight; SE = standard error; X² = Chi square; df = degrees of freedom; *p* = significance; OR = odds ratio; CI = confidence interval

Regarding PA in sport (club) participation, binary logistic regression revealed statistically significant results for disability as well as all confounders (Table 4). If children have a disability, they are less likely to participate in sport (OR: 0.55, 95% CI [0.42, 0.71]) as well as in sport club (OR: 0.59, CI [0.45, 0.78]).

## Discussion

The present study aimed to identify the PA participation in children and adolescents with a large variety of different disabilities focussing on the fulfilment of PA recommendations as well as participation in sporting activities and sport clubs. Further, differences and similarities regarding these PA related health behaviours in CAWD and CAWoD have been identified.

### Compliance with PA recommendations

Regarding the compliance with the WHO PA recommendations our analysis revealed no statistically significant differences between CAWD and CAWoD. Both values are on an alarming low level, indicating that less than or roughly one quarter of CAWD and CAWoD, respectively, accumulate enough PA during their everyday life. As regularly meeting the PA levels that are recommended, is associated with numerous health effects [37], the promotion of PA should be a high priority in public health policies. Further, the effect estimates are indicating that the age, sex as well as residential are associated with the fulfilment. With increasing age, the likelihood of meeting the WHO PA recommendations decreases. Further, boys are more likely than girls to meet the WHO PA recommendations. In addition, children/adolescents living in rural areas are more likely to be physically active than in cities. However, these findings have also been confirmed in other studies [38]. Although the overall levels of compliance with the WHO PA recommendations did not significantly differ between CAWD and CAWoD, disparities in sport and club participation point toward important qualitative differences in how PA is achieved. It is possible that CAWD accumulate PA through less structured or informal means (e.g., unstructured play, home-based activity), while CAWoD more often participate in organized sports and clubs. These divergent pathways to reaching PA guidelines may reflect underlying structural inequalities rather than equivalence in opportunity or experience. In this sense, the absence of a statistical difference in WHO compliance should not be misinterpreted as equal access or inclusion.

Additionally, gender and age appear to moderate these patterns: older children and girls were less likely to meet PA recommendations, echoing trends in previous studies {Craggs, 2011 #22069}. This highlights that disability does not operate in isolation. The intersection with other social determinants, such as age, gender, and environment, further shapes participation—and should be addressed in future analyses and policy.

Nevertheless, in contrast to the sport outcomes in case of the PA recommendations no statistically significant differences with regards to disabilities were observed. This suggests that, in terms of overall daily physical activity, children and adolescents with disabilities may reach similar activity levels as their peers without disabilities. However, this finding does not necessarily indicate equal access or absence of barriers. It is possible that CAWD engage more in unstructured or non-organized forms of PA, such as home-based activities, therapeutic movement, or active play, while facing barriers to participating in organized or structured sport. This interpretation aligns with previous research, which presents a mixed picture: while the European cross-national Health Behaviour in School-aged Children (HBSC) study (2013–14) similarly found no statistically significant differences in meeting PA recommendations between boys or girls with and those without long-term illnesses or disabilities at a national level [39], other studies highlight structural and environmental barriers limiting PA participation among CAWD [40, 41]. Thus, both can be true at the same time: CAWD may face significant challenges in access to structured PA, yet still achieve the recommended amount of overall physical activity through alternative means. While some studies have already investigated PA behavior in children with specific disabilities or in selected regions of Germany, future research should expand on this by including broader samples, a greater variety of disability types, and diverse PA settings in order to provide a more nuanced understanding of potential disparities.

### Sport club participation

Further, regarding participation in sport clubs, 44.6% of CAWD participate in sport clubs. These statistically significant results in sport club participation indicate lower participation chances in CAWD which indicates a limited inclusion. This disadvantage of CAWD is also indicated by several international studies. CAWD are confronted with several barriers when participating in sport club activities such as missing opportunities as well as missing acceptance [42]. Moran and Block [43] indicated barriers such as a lack of knowledge of coaches regarding the needs of CAWD. Further, finding an appropriate sport club can be challenging for CAWD and their parents [16]. In addition, only a few sport clubs have opportunities for participation of CAWD [44]. To increase PA participation in sport clubs, the specific barriers for people with disabilities should be identified and reduced.

### Sport activities

Across all participants of the KiGGS study, 60.4% of CAWD participate in sports. As in the present study sports activities were defined as all sporting activities except of physical education at school or in nursery, this includes active play and also sport club participation. The Global Matrix of Para Report Cards indicated a low participation of CAWD in active play as on average only 30% met the benchmarks [10]. Further, in organised sport and PA the Global Matrix of Para Report Cards indicated a participation of 20 to 26% of CAWD [10]. These values are consistent with our results. CAWD are less likely to participate in sports than CAWoD. This may be due to several barriers, CAWD are confronted with when participating in sports. Social and environmental barriers such as the acceptance of peers or limited mobility prevent children and adolescents being active in sports [14]. Further, a lack of nearby facilities or programs for sport activities for CAWD was also indicated as possible barriers in previous studies [45]. Therefore, it is necessary to reduce barriers for CAWD and to increase participation. Identifying further barriers and facilitators to sports activities and promoting sports participation in CAWD should be focused in further research.

### Strength & limitations

The study analyses the PA behaviour of CAWD and compares their values with CAWoD. This comparison helps to obtain the necessary data for future research and to promote PA in all children and adolescents. To the best of our knowledge this is the first German study analysing and comparing CAWD and CAWoD PA behaviour in a large national sample.

Nevertheless, there are several limitations that should be stated. First, the sample size of CAWD (*N* = 271) is relatively small, which may limit the statistical power and generalizability of the findings. This should be considered when interpreting the results. A smaller sample size can reduce statistical power, increasing the likelihood of Type II errors, meaning that potential differences may not reach statistical significance despite their real-world relevance. Moreover, the limited sample size may affect the generalizability of the findings, as it may not fully capture the diversity of PA behaviors among CAWD. Future studies should aim for larger and more diverse samples to provide more robust and representative insights into PA participation in this population. Additionally, future studies should consider combining self-reported data with objective measurement tools such as pedometers or accelerometers to enhance the validity of PA data. While self-report instruments are practical and cost-effective, especially for large and diverse populations, they are prone to recall and social desirability bias, which can lead to over- or underestimation of physical activity levels. Further, the present study focuses on the prevalence of PA but does not capture the amount or intensity of PA, which could provide a more detailed understanding of activity patterns. Furthermore, it must be noted that in the present study, the measure of “sporting activities” includes activities performed within sport clubs. This overlap between the two indicators may lead to double counting, as children and adolescents who participate in sport clubs were also likely to report general sport participation. While the separation between structured (e.g., club-based) and unstructured sport was not fully possible based on the available questions, future research should aim to distinguish more clearly between organized and informal physical activity settings to better understand where and how children engage in sports. Fourth, no distinctions between different types of disabilities were made, meaning potential differences in PA behavior among CAWD with various disabilities remain unexamined. Additionally, other relevant factors influencing PA participation, such as environmental barriers, social support, or access to inclusive sports programs, were not assessed. Future research should consider these aspects to gain a more comprehensive picture of PA behavior in CAWD.

## Conclusion

The present study provided new insights in the PA behaviour of CAWD. In comparison to CAWoD, CAWD are disadvantaged in their PA behaviour. Although the fulfilment of the WHO-guideline is comparable in CAWD and CAWoD the present study indicated statistically significant results regarding participation in sporting activities as well as in sport clubs. Therefore, there is a lack of inclusion and participation in their recreational time. This disadvantage may be due to several barriers CAWD are confronted with when they want to participate. Future research in the PA behaviour of CAWD should analyse these determinants and could provide information on how PA can be promoted, especially in CAWD.

## Data Availability

The datasets used and/or analysed during the current study are available from the corresponding author on reasonable request.
